# Excess Mortality During the COVID-19 Pandemic in Sonora, Mexico, 2015–2022: A Time-Series Analysis of Cause-Specific Mortality

**DOI:** 10.3390/epidemiologia7040105

**Published:** 2026-07-28

**Authors:** Diego I. Álvarez-López, Elizabeth Ferreira-Guerrero, Lina S. Palacio-Mejía, Amado D. Quezada-Sánchez, Jorge Laureano-Eugenio, Sergio Trujillo-López, Mariann Duarte-Badillo, Gerardo Álvarez-Hernández

**Affiliations:** 1 Department of Population and Public Health Sciences, Keck School of Medicine, University of Southern California, Los Angeles, CA 90032, USA; dialvare@usc.edu; 2 Centro de Investigación Sobre Enfermedades Infecciosas, Instituto Nacional de Salud Pública, Cuernavaca 62100, Mexico; elizabeth.ferreira@insp.mx; 3 Centro de Investigación en Evaluación y Encuestas, Instituto Nacional de Salud Pública, Cuernavaca 62100, Mexico; lpalacio@insp.mx (L.S.P.-M.); amado.quezada@insp.mx (A.D.Q.-S.); 4 Subsecretaría de Servicios de Salud Colectiva, Secretaría de Salud Pública del Estado de Sonora, Hermosillo 83000, Mexico; subserviciossaludcolectiva@gmail.com; 5 Departamento de Medicina y Ciencias de la Salud, Universidad de Sonora, Hermosillo 83000, Mexico; sergio.trujillo@unison.mx (S.T.-L.); a222214072@unison.mx (M.D.-B.)

**Keywords:** excess mortality, time-series analysis, public health surveillance, mortality trends, COVID-19, Mexico

## Abstract

**Background/Objectives**: Excess mortality (EM) is a key indicator for assessing the population-level impact of large-scale health crises, particularly when cause-of-death ascertainment is incomplete or delayed. However, EM estimates are sensitive to methodological decisions, highlighting the need for operationally feasible approaches suitable for routine public health surveillance. **Methods**: We conducted time series analyses of routinely collected mortality data from a subnational setting in northern Mexico. Deaths recorded between 2015 and 2022 were grouped into 28 cause-of-death categories. Expected deaths during the COVID-19 pandemic period (March 2020–July 2022) were estimated using negative binomial regression models fitted to pre-pandemic data (2015–2019), incorporating a linear trend and monthly indicators to account for long-term trends and seasonality. Newey–West standard errors were used to address serial correlation and heteroskedasticity. EM was defined as the difference between observed and expected deaths. **Results**: An estimated 14,482 excess deaths were observed during the pandemic period, corresponding to a 30.9% increase relative to expected mortality. Time-series models identified four distinct peaks of excess mortality coinciding with major pandemic waves. Although COVID-19 accounted for most excess deaths, among non-COVID causes, only ischemic heart disease and diabetes showed statistically significant excess mortality among major non-communicable diseases after baseline adjustment. However, additional categories—including non-transport-related accidents, ill-defined causes, and other endocrine, metabolic, hematological, and immunological diseases—also exhibited statistically significant excess mortality. For several causes, increases in crude mortality did not translate into statistically significant excess mortality. **Conclusions**: Negative binomial time series regression provides an implementable framework for estimating EM, underscoring the importance of expected mortality estimation for understanding population-level mortality dynamics during health emergencies.

## 1. Introduction

An epidemic is defined as the occurrence of a disease or health event at a frequency exceeding the expected baseline within a population, whereas a pandemic denotes its widespread, often global, propagation, typically accompanied by substantial morbidity and mortality [1]. Central to both concepts is the comparison between observed and expected health events over time. The COVID-19 pandemic profoundly disrupted health systems and societies worldwide, posing major challenges for accurately quantifying its mortality impact, particularly beyond deaths directly attributed to this disease.

In this context, excess mortality (EM) has emerged as an integrative indicator for assessing the population-level impact of large-scale health crises. The World Health Organization (WHO) estimated that between 13.2 and 16.6 million excess deaths occurred globally between January 2020 and December 2021, reflecting both direct and indirect impacts of the pandemic [2,3]. EM is especially informative in settings where cause-of-death ascertainment may be incomplete, delayed, or heterogeneous; however, its estimation is highly sensitive to underlying methodological choices used to define expected mortality [4].

Countries experienced substantial heterogeneity in EM during the COVID-19 pandemic, shaped by demographic structure, socioeconomic conditions, health system capacity, and data quality [2,3]. Low- and middle-income countries were disproportionately affected, often facing additional challenges related to limitations in mortality registration systems and cause-of-death certification [5,6]. These constraints further underscore the importance of analytically robust yet operationally feasible methods for estimating EM at both national and subnational levels.

EM is defined as the difference between observed and expected deaths over a given period and captures both increases and decreases in cause-specific mortality. Unlike cause-based COVID-19 mortality counts, EM can reflect the net impact of a pandemic, including deaths indirectly attributable to health system disruption, delayed care, and broader social and economic consequences. However, EM estimates are highly sensitive to methodological decisions, including baseline selection, model specification, handling of seasonality and long-term trends, and the choice of statistical distribution for mortality counts [7]. Careful consideration of these elements is therefore essential to ensure valid interpretation.

During the COVID-19 pandemic, a wide range of statistical approaches were used to estimate EM, developed by academic groups [8] and public health institutions such as the WHO [9] and the U.S. Centers for Disease Control and Prevention [10]. This methodological heterogeneity reflects differences in analytical objectives, data availability, and trade-offs between model complexity, transparency, and interpretability. Although increasingly complex modeling strategies have been proposed, time-series regression models for count data remain widely used in epidemiologic surveillance due to their interpretability, robustness, and applicability in routine public health settings, particularly for mortality surveillance [11,12].

Despite their widespread use, practical illustrations of how negative binomial time series models can be applied to estimate both all-cause and cause-specific EM in real-world, subnational contexts remain relatively scarce in published studies from Latin American settings. In this study, we aim to both estimate EM in a subnational setting and illustrate key methodological considerations in its estimation using routinely collected surveillance data. Specifically, we use data from Sonora, Mexico, a northern border state with distinctive demographic dynamics and health system characteristics, as a case study to demonstrate how a parsimonious and operationally feasible modeling approach can be applied in real-world public health surveillance contexts, while also providing empirically grounded insights into pandemic-related mortality patterns.

## 2. Materials and Methods

### 2.1. Data Sources and Study Design

Time-series analyses were conducted to illustrate key methodological considerations in estimating EM using negative binomial regression models applied to routinely collected mortality surveillance data. The analyses examined leading causes of mortality and their variation relative to expected deaths in the state of Sonora, Mexico, from 1 March 2020 to 31 July 2022.

The study included all deaths recorded during the study period in the Epidemiological and Statistical Death Subsystem (SEED) of the Sonora Health Services, as well as deaths from the five preceding pre-pandemic years (2015–2019), which were used to establish baseline mortality patterns. SEED is a nationwide technological platform that compiles preliminary information from death certificates for epidemiological surveillance, health service evaluation, and the production of mortality statistics [13]. The system captures information on medical care received, characteristics of the death, sociodemographic attributes of decedents, and cause-of-death coding based on the International Classification of Diseases, 10th Revision (ICD-10) [14].

Because SEED is a secondary dataset derived from Mexico’s national mortality surveillance system rather than a research-specific data collection process, the analyses reflect the constraints and opportunities inherent to real-world public health surveillance data. All analyses relied on the variables and ICD-10 coding conventions established for routine mortality statistics. SEED provides complete statewide coverage, standardized cause of death certification, and consistent temporal reporting, features that support its suitability for modeling all-cause and cause-specific mortality over time. No transformations beyond standard epidemiologic aggregation were applied. Data limitations intrinsic to secondary surveillance systems (e.g., lack of control over primary data collection) were addressed through explicit and transparent methodological specification.

### 2.2. Cause-of-Death Classification

COVID-19 deaths were defined using ICD-10 codes U07.1 and U07.2 and analyzed as a separate cause-of-death category. All non-COVID cause-of-death categories exclude deaths coded as COVID-19, ensuring mutually exclusive classification based on the recorded underlying cause of death. Given that COVID-19 did not exist as a certified cause of death prior to the pandemic period, expected deaths for this category were assumed to be zero. This specification reflects the absence of pre-pandemic baseline mortality for COVID-19 and should be considered when comparing this category with other causes modeled using pre-pandemic data.

Coded deaths were grouped into 22 cause-of-death categories following the classification proposed by the 2019 Global Burden of Disease (GBD) study [15]. Given their epidemiologic relevance in the study setting, cardiovascular diseases and injuries were further stratified into four and two subgroups, respectively. Injury-related deaths were specifically divided into transport-related and non-transport-related categories, resulting in a total of 28 groups and subgroups [16]. This classification strategy was selected to explore heterogeneity in EM across major causes while maintaining interpretability for surveillance-oriented analyses.

### 2.3. Analytical Periods and Stratification

For modeling purposes, the baseline period was defined as January 2015 through February 2020. However, January–February 2020 was not included in the descriptive analyses. Mortality was stratified by sex, age group, health district of residence, employment status, cause of death, medical care received, place of death, education level, and entitlement to health services.

Annual mortality rates (MRs) were calculated for each of the six Health Districts (HDs) and for the state overall using death counts and population estimates from both periods. Rates were standardized per 1000 population and averaged annually to facilitate comparisons across periods of unequal duration. Annual average death counts and crude MRs were computed for each of the 28 cause categories. During the pandemic period, deaths were classified as either attributable to COVID-19 or to other causes. All registered deaths were included.

### 2.4. Statistical Modeling and Estimation of Excess Mortality

All statistical models were fitted using monthly aggregated death counts, which defined the analytical time scale of the study. Expected monthly deaths during the pandemic period were estimated using negative binomial regression models with a log link fitted to each cause-specific and subgroup time series using pre-pandemic data from 2015 to 2019. This baseline period was selected to reflect stable pre-pandemic mortality trends unaffected by COVID-19-related disruptions.

The models incorporated Newey–West standard errors (two lags were specified following the rule of thumb of using the integer part of T^1/4^ for the number of lags) to improve robustness to heteroskedasticity and serial autocorrelation commonly observed in monthly mortality time series [17]. The linear predictor included a secular linear trend and monthly dummy variables to account for long-term trends and seasonal variation. This specification provides a parsimonious and interpretable representation of monthly mortality time series in applied settings. We also explored using a linear spline with two knots at the 40th and 60th percentile for the secular trend in the linear predictor [16]. Only the cause category of events of undetermined intent showed a better fit with this specification, as indicated by the Akaike and Bayesian information criteria (Supplementary Material, Table S1); we used this type of secular trend only for this group.

Model parameters estimated from the pre-pandemic period were used to project the counterfactual expected number of monthly deaths during the pandemic period in the absence of COVID-19. Expected cumulative deaths were obtained by summing projected monthly estimates across the pandemic period. Standard errors for the expected cumulative deaths were derived using the Delta method [18]. These standard errors were used to calculate 95% confidence intervals, except for the events of undetermined intent group for which our confidence interval was derived for the logarithm of the expected cumulative deaths and back-transformed to avoid a negative confidence lower bound.

Excess deaths were calculated as the difference between observed and expected cumulative deaths. Percentage change in cumulative EM was computed using the formula: [(cumulative observed deaths/cumulative expected deaths) − 1] × 100.

Expected deaths for all-cause mortality and for cardiovascular diseases were estimated by summing model-generated expected deaths across the relevant cause groups. Associated standard errors were obtained by taking the square root of the sum of squared standard errors across component cause groups. This strategy provides consistency so that the sum of estimated expected deaths over a group of causes matches the estimated total of expected deaths; however, it assumes independence between component cause-specific series. As an additional analysis, we performed our analysis on the series of total deaths and the group of cardiovascular disease deaths, which does not require the independence assumption. All analyses were conducted using Stata/MP 19.0.

## 3. Results

Between 1 January 2015 and 31 December 2022, a total of 162,779 deaths were recorded in Sonora; 61,293 (37.7%) occurred during the pandemic period, of which 12,528 (20.4%) were certified as COVID-19. The demographic pattern remained stable across periods, with most deaths among males and adults aged 65 years and older. Patterns in healthcare differed: Among COVID-19 decedents, 90.7% received medical care prior to death—substantially higher than for non-COVID causes in the pre-pandemic (77.8%) and pandemic (77.4%) periods; 90.4% of COVID-19 deaths occurred in medical facilities. In contrast, the proportion of non-COVID-19 deaths that occurred in medical facilities declined from 56.1% before the pandemic to 47.9% during the pandemic. Violent deaths comprised a larger share during the pandemic (8.4%) than before (5.1%) (Table 1).

**Figure 1:**
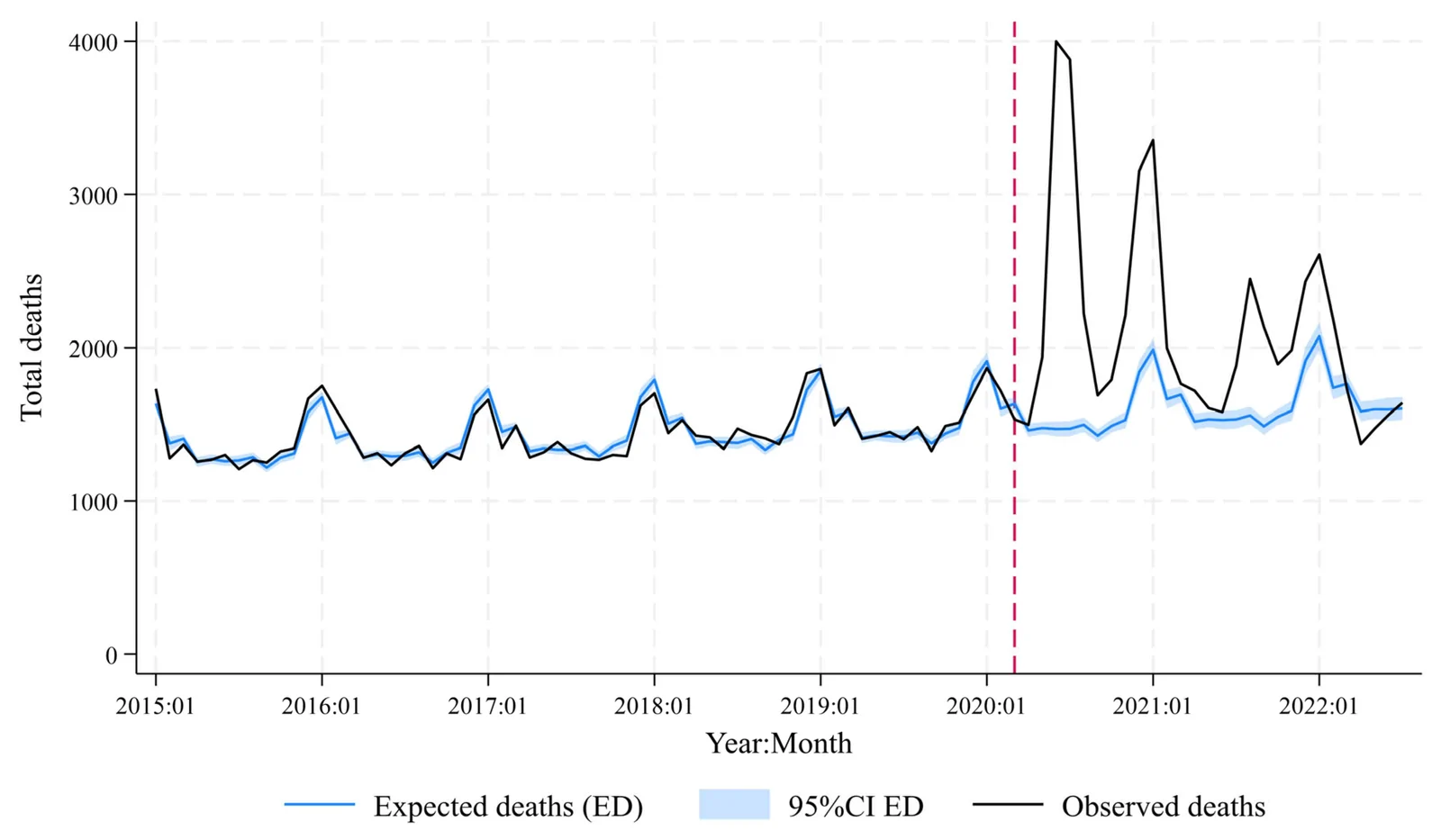
Time series of all-cause mortality in Sonora, Mexico, 2015–2022. The vertical dashed red line marks the starting month of the COVID-19 pandemic in Mexico (March 2020).

**Figure 2:**
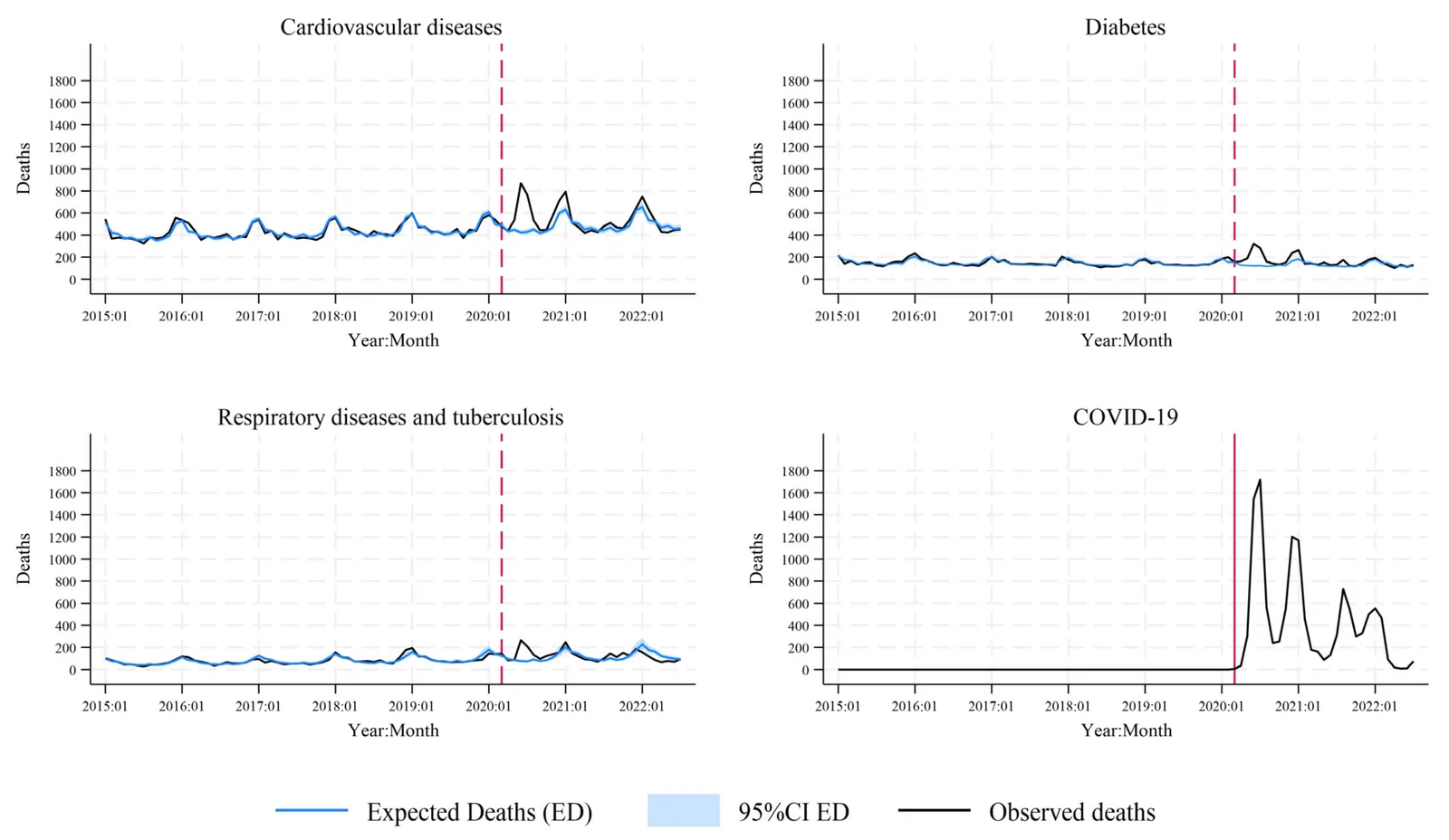
Monthly trends in observed and expected deaths for leading causes of excess mortality in Sonora, Mexico, 2015–2022. Each panel displays observed deaths (black line), model-based expected deaths (blue line), and 95% confidence intervals (shaded blue area). The vertical dashed red line marks the starting month of the COVID-19 pandemic in Mexico (March 2020).

Time-series negative binomial models identified four distinct peaks of excess mortality (EM) corresponding to major pandemic waves: mid-June 2020, early January 2021, mid-August 2021, and early January 2022. These peaks were identified based on visual comparison of observed and expected mortality trends over time (Figure 1 and Figure 2). The first peak coincided with increases in cardiovascular diseases, diabetes, and respiratory diseases/tuberculosis, illustrating how the modeling approach captures temporal clustering of cause-specific mortality relative to an expected seasonal trend baseline.

At the population level, the crude mortality rate (MR) rose 39.7%, from 5.49 to 7.67 per 1000 population from the pre-pandemic to the pandemic period. All six Health Districts (HDs) exhibited similar upward trends (Table 2), showing broad-based increases. These patterns are consistent with multiple potential factors, including changes in population dynamics, epidemic timing, and healthcare access. Regional variation is reported to illustrate how the approach accommodates heterogeneity within a routine surveillance system, although detailed HD-level patterns are not the primary focus of this methodological application.

The EM analysis estimated 14,482 excess deaths (95% CI: 13,263–15,701) during the pandemic, corresponding to a 30.9% increase (95% CI: 27.6–34.4%) over expected deaths. COVID-19 accounted for the majority of excess deaths; among non-COVID causes, ischemic heart disease (12.4%) and diabetes (6.4%) contributed most. When examined as percentage excess relative to expected deaths (%EM), only ischemic heart disease (+17.5%, 95% CI: 12.1–23.5) and diabetes (+21.8%, 95% CI: 14.0–30.7) showed statistically significant EM. No significant %EM was observed for respiratory diseases and tuberculosis, homicides, genitourinary diseases, or mental and nervous system disorders, or maternal diseases, the latter showing wide confidence intervals consistent with a small number of deaths. Significant reductions in EM were identified for non-infectious respiratory diseases (−21.5%, 95% CI: −30.7 to −9.6), and infectious and parasitic diseases (−13.0%, 95% CI: −19.9 to −4.9). In addition, statistically significant EM was observed for non-transport-related accidents (+22.7%, 95% CI: 12.0–35.7), ill-defined causes (+50.7%, 95% CI: 25.9–87.7), and other endocrine, metabolic, hematological, and immunological diseases (+26.0%, 95% CI: 11.7–44.4), indicating broader variation in cause-specific mortality patterns beyond the main categories (Table 3). Our model applied to cardiovascular diseases and total deaths time series, as an alternative to obtaining these estimates from the individual cause subgroups analyses, showed similar results. EM = 1570 (95% CI: 1079–2060) and EM% = +11.2% (95% CI: 7.4–15.2) for cardiovascular diseases and EM = 14,920 (13,753– 16,087) and EM% = 32.2 * (95% CI: 28.9–35.6) for all causes. Plots of expected and observed deaths for individual series are shown in the Supplementary Material (Figures S1–S7).

Across causes, differences between crude MR and model-based EM estimates were observed. In several instances, increases in crude mortality did not correspond to statistically significant EM after adjustment for baseline trends and seasonality, while some categories with significant EM were less apparent in crude comparisons. These patterns illustrate the contribution of baseline-adjusted time-series modeling to the interpretation of pandemic-period mortality.

## 4. Discussion

This study illustrates the application of negative binomial time series regression to estimate all-cause and cause-specific EM using routinely collected mortality surveillance data in a subnational setting. From 1 March 2020 to 31 July 2022, we estimated a 30.9% all-cause EM, equivalent to 13,332 deaths, relative to a pre-pandemic baseline (2015–2019). The magnitude of EM falls within the broadly reported range for the COVID-19 period [16,19,20]. However, cross-setting comparisons should be interpreted cautiously given differences in baseline selection, data completeness, cause-of-death attribution, and modeling choices [4,5,7].

A central empirical insight from this application is that crude mortality rates (MR) and model-based EM do not always converge across causes. Whereas MR captures overall changes in mortality volume during the pandemic, EM identifies departures that are statistically significant after accounting for long-term trends and seasonality [9,21]. This distinction is evident in the results shown in Table 3. For example, some cause-of-death categories exhibited increases in crude mortality during the pandemic period but did not show statistically significant EM after adjustment. Conversely, certain categories with statistically significant EM—such as non-transport-related accidents, ill-defined causes, and other endocrine, metabolic, hematological, and immunological diseases—were less apparent in crude comparisons.

In this case study, the time-series models identified four distinct peaks of EM aligned with major pandemic waves, reflecting temporal deviations from expected patterns in routinely collected surveillance data [22,23]. These findings are consistent with previously documented patterns of mortality disruption during the COVID-19 pandemic, although the specific mechanisms underlying these changes cannot be directly evaluated within the present ecological time-series framework. Increases in mortality across settings may therefore reflect a combination of factors, including differences in population structure, exposure patterns, epidemic timing, baseline morbidity, and access to care.

Cause-specific analyses showed that COVID-19 accounted for most excess deaths, as observed in multiple settings [3,8,9,22,23]. Among non-communicable diseases, only a subset—particularly ischemic heart disease and diabetes—exhibited significant EM once expected variation was incorporated, aligning with individual-level evidence of higher mortality risk in persons with cardiometabolic conditions and with population-level reports of increased EM from cardiovascular disease and diabetes during the pandemic [24,25,26,27]. By contrast, significant reductions in EM for non-infectious respiratory diseases, neonatal conditions, and infectious and parasitic diseases may be consistent with indirect effects of non-pharmaceutical interventions (e.g., masking, physical distancing, and improved hygiene) on the transmission of respiratory and other communicable pathogens, as well as potential shifts in health-seeking behavior [28,29].

However, these patterns may also reflect alternative explanations, including cause-of-death displacement, changes in death certification practices, delays in death registration, reduced diagnostic workup, and competing mortality risks related to COVID-19. Importantly, EM does not disentangle mechanisms; therefore, the relative contribution of these factors—including changes in incidence, healthcare disruption, or care pathway shifts—cannot be resolved within the present ecological time-series design [21].

An additional consideration in the interpretation of cause-specific EM relates to potential misclassification of deaths during the pandemic period. Changes in death certification practices, limited diagnostic confirmation—particularly during peak periods of health system demand—and shifts in coding priorities may have led to misclassification of some COVID-19 deaths into other cause-of-death categories, including respiratory, cardiovascular, and ill-defined causes. Because cause-specific analyses rely on the recorded underlying cause of death, such misclassification may influence apparent increases or decreases in certain categories. These patterns should therefore be interpreted with caution, as they may partly reflect differences in certification practices and diagnostic capacity rather than true changes in underlying disease occurrence.

In particular, the observed increase in ill-defined causes is especially relevant for interpreting cause-specific EM patterns. Such increases may vary geographically and could reflect changes in death certification practices, reduced access to diagnostic confirmation—especially during peak periods of health system demand—and broader challenges in mortality surveillance quality during the pandemic. These factors may affect the interpretation of cause-specific EM by potentially redistributing deaths across diagnostic categories. In this context, increases in ill-defined causes may partly reflect diagnostic uncertainty or incomplete attribution, which could lead to underestimation or overestimation of EM in specific cause-of-death groups.

Regional heterogeneity in mortality patterns is expected and has been documented in diverse contexts, reflecting differences in demographic composition, baseline health status, access to and quality of care, timing and stringency of public health measures, and the distribution of high exposure occupations [23,30,31,32]. In our study, such variation is presented primarily to illustrate that the modeling framework can accommodate within-region differences using routine surveillance data; detailed spatial attribution is beyond the scope of this methodological application.

A key strength of this study is the use of a transparent, parsimonious modeling framework that can be implemented using standard mortality data structures, balancing interpretability and robustness for routine public health surveillance. This approach supports reproducibility and enables timely monitoring in settings where data completeness and computational resources may be constrained.

Limitations are inherent to retrospective analyses of routinely collected mortality data. Uncertainty in population denominators may affect the precision of crude mortality estimates, particularly in the context of migration and demographic changes during the pandemic period. In addition, delays in death registration and variability in reporting completeness may have influenced the temporal alignment between observed and expected deaths.

Cause-of-death misclassification is an additional source of uncertainty. Changes in death certification practices, limited diagnostic confirmation—especially during periods of health system demand—and broader challenges in surveillance data quality may have affected the consistency of cause-of-death attribution, potentially redistributing deaths across diagnostic categories and influencing apparent changes in specific causes of death.

Further methodological considerations relate to model specification and analytical choices. The selection of the baseline period, specification of a linear secular trend with monthly indicators, the use of Newey–West standard errors to address serial correlation, and the derivation of cumulative uncertainty using the Delta method can influence expected deaths and their associated confidence intervals [17,18]. These decisions were prespecified to align with routine public health use cases rather than to maximize statistical complexity [33]. When aggregating uncertainty across cause-of-death categories, the calculation assumes independence between component series; in practice, correlations across causes may influence variance estimates. However, compared to analyzing the aggregated subgroups separately, the widths of confidence intervals were slightly more conservative in our main analysis and differed only by +0.1 percentage points for total EM% and by +0.5 percentage points for cardiovascular diseases EM%.

Sensitivity analyses using alternative baseline periods or model structures may provide additional insight into the robustness of EM estimates; however, such analyses were beyond the scope of this study, which prioritizes transparency and operational feasibility [21,33,34].

Finally, the ecological time-series design limits the ability to infer causal mechanisms at the individual level. Observed patterns should therefore be interpreted as population-level phenomena, and the relative contribution of competing mechanisms underlying mortality changes cannot be disentangled within this framework.

As for implications for surveillance and practice, Poisson time-series models provide an operational and scalable framework to quantify EM using standard mortality surveillance systems, including in contexts with delayed or incomplete data [20,21,22]. Reporting both crude mortality rates and model-based EM is informative, as the former captures overall changes in mortality volume whereas the latter identifies statistically significant deviations from expected patterns after accounting for seasonality and long-term trends [35]. Strengthening analytic capacity to implement such approaches within surveillance systems may improve anomaly detection, support timely preventive responses, and enhance preparedness for future public health emergencies.

## 5. Conclusions

This case study demonstrates how negative binomial time series regression can be used to estimate EM using routinely collected data, offering an operationally feasible and interpretable approach for public health surveillance. By showing that EM and crude mortality rates may diverge once expected variation is accounted for, our study highlights an important methodological consideration: estimating expected mortality is central to understanding population-level mortality dynamics during pandemics and other large-scale health crises, particularly when cause-of-death ascertainment is incomplete or delayed.

These findings should be interpreted within the context of the data and methodological limitations discussed and underscore the value of combining robust analytical approaches with cautious interpretation in routine mortality surveillance.

## Figures and Tables

**Table 1:** Sociodemographic characteristics of deaths, 2015–2022, Sonora, Mexico.

Variable	2015–2019 ^#^ (Pre-Pandemic)	2020–2022 * (Pandemic)	
	All Causes (*n* = 88,367)	COVID-19 (*n* = 12,528)	Other Causes (*n* = 48,765)
	*N* (%)	*N* (%)	*N* (%)
Sex			
Male	52,164 (59.03)	7191 (57.40)	29,249 (59.98)
Female	35,989 (40.73)	5323 (42.49)	19,321 (39.62)
Not known	214 (0.24)	14 (0.11)	195 (0.40)
Age group (years)			
<20	4708 (5.33)	36 (0.29)	1911 (3.92)
20–44	11,780 (13.33)	1160 (9.26)	7388 (15.15)
45–64	22,634 (25.61)	4422 (35. 30)	12,606 (25.85)
65 and older	48,724 (55.14)	6907 (55.13)	26,621 (54.59)
Not known	521 (0.60)	3 (0.02)	239 (0.49)
Education level			
None	10,490 (11.87)	851 (6.79)	4233 (8.68)
Basic ^&^	60,771 (68.77)	7861 (62.75)	32,535 (66.72)
High school	7379 (8.35)	1373 (10.96)	4594 (9.42)
Graduates	4120 (4.66)	1488 (11.88)	3433 (7.04)
Not applicable (<4 years old)	1905 (2.16)	8 (0.06)	990 (2.03)
Not known	3702 (4.19)	947 (7.57)	2980 (6.11)
Entitlement to health services			
Yes	72,964 (82.57)	9952 (79.44)	33,341 (68.37)
No	9062 (10.25)	1786 (14.26)	10,226 (20.97)
Not known	6341 (7.18)	789 (6.30)	5198 (10.66)
Employed			
Yes	17,152 (19.41)	2960 (23.63)	10,148 (20.81)
No	64,664 (73.18)	8257 (65.91)	35,082 (71.94)
Not known	6551 (7.41)	1312 (10.47)	3535 (7.25)
Health District			
I. Hermosillo	26,264 (29.72)	4186 (33.41)	14,049 (28.81)
II. Caborca	3362 (3.80)	362 (2.89)	1965 (4.03)
III. Santa Ana	11,965 (13.54)	1671 (13.34)	6720 (13.78)
IV. Ciudad Obregón	23,829 (26.97)	3390 (27.06)	13,635 (27.96)
V. Navojoa	12,300 (13.92)	1455 (11.61)	6461 (13.25)
VI. San Luis Río Colorado	6748 (7.64)	1040 (8.30)	4077 (8.36)
Foreign	2231 (2.52)	400 (3.19)	1375 (2.82)
Not known	1668 (1.89)	25 (0.20)	483 (0.99)
Cause of death			
Disease	77,769 (88.00)	12,528 (100)	42,147 (86.43)
Accident	3796 (4.30)	0	1785 (3.66)
Violent	4555 (5.16)	0	4111 (8.43)
Not known	2247 (2.54)	0	722 (1.48)
Medical care prior to death			
Yes	68,730 (77.78)	11,363 (90.70)	37,749 (77.41)
No	15,566 (17.62)	242 (1.93)	8344 (17.11)
Not known	4071 (4.61)	923 (7.37)	2672 (5.48)
Place of death			
Medical unit	49,661 (56.20)	11,320 (90.36)	23,354 (47.89)
Public road	5269 (5.96)	33 (0.26)	3340 (6.85)
Home	27,733 (31.38)	1011 (8.07)	18,053 (37.02)
Other	5299 (6.00)	137 (1.09)	3667 (7.52)
Not known	405 (0.46)	28 (0.22)	351 (0.72)

^#^ 1 January 2015–31 December 2019; * 1 March 2020–31 July 2022; ^&^ Refers to elementary and lower secondary education.

**Table 2:** Mortality rates by Health District in Sonora, Mexico, comparing the pre-pandemic (2015–2019) and pandemic (2020–2022) COVID-19 periods.

Health District	2015–2019 ^#^	2020–2022 *	% Change ^&^
	M.R. ^1/^	M.R. ^1/^	
Hermosillo (I)	5.19	7.16	37.9
Caborca (II)	5.48	7.67	39.9
Santa Ana (III)	4.86	6.83	40.5
Ciudad Obregón (IV)	6.28	8.98	42.9
Navojoa (V)	6.30	8.14	29.2
San Luis Río Colorado (VI)	4.53	7.47	64.9
State of Sonora	5.49	7.67	39.7

^1/^ Annual standardized mortality rates per 1000 population; ^#^ January 2015–December 2019. * March 2020–July 2022. ^&^ Percentage change from the pre-pandemic to the pandemic period. Source: General Directorate of Health Information (DGIS), Ministry of Health. Data Cube: Population Projections by Health Service Entitlement Status. Note: Estimates by DGIS based on population projections produced by the National Population Council (CONAPO, 2018) https://www.gob.mx/conapo/documentos/bases-de-datos-de-la-conciliacion-demografica-1950-a-2019-y-proyecciones-de-la-poblacion-de-mexico-2020-a-2070, accessed: 4 August 2023.

**Table 3:** Differences between observed deaths and expected deaths (March 2020–July 2022), Sonora, Mexico.

Cause of Death Subgroup	Expected Deaths (95% CI)	Observed Deaths †	Excess Deaths (95% CI)	% of Excess Deaths (95% CI)
COVID-19	0	12,528	12,528	-
Cardiovascular disease	14,103 (13,576, 14,631)	15,637	1534 * (1006, 2061)	10.9 * (6.9, 15.2)
—Ischemic heart disease	9134 (8691, 9577)	10,734	1600 * (1157, 2043)	17.5 * (12.1, 23.5)
—Cerebrovascular disease	1964 (1842, 2086)	1976	12 (−110, 134)	0.6 (−5.3, 7.3)
—Hypertensive heart disease	551 (473, 629)	555	4 (−74, 82)	0.8 (−11.7, 17.4)
—Other cardiovascular diseases	2455 (2209, 2701)	2372	−83 (−329, 163)	−3.4 (−12.2, 7.4)
Diabetes	3873 (3608, 4137)	4717	844 * (580, 1109)	21.8 * (14.0, 30.7)
Respiratory infections and tuberculosis	3374 (2887, 3861)	3650	276 (−211, 763)	8.2 (−5.5, 26.4)
Non-transport-related accidents	1433 (1296, 1570)	1758	325 * (188, 462)	22.7 * (12.0, 35.7)
Ill-defined causes	372 (299, 446)	561	189 * (115, 262)	50.7 * (25.9, 87.7)
Other endocrine, metabolic, hematological, and immunological diseases	417 (364, 471)	526	109 * (55, 162)	26.0 * (11.7, 44.4)
Transport-related accidents	1050 (918, 1182)	1104	54 (−78, 186)	5.1 (−6.6, 20.2)
Genitourinary diseases	1175 (1039, 1311)	1223	48 (−88, 184)	4.1 (−6.7, 17.7)
Congenital defects	315 (251, 379)	325	10 (−54, 74)	3.1 (−14.2, 29.2)
Maternal diseases	11 (5, 17)	16	5 (−1, 11)	43.9 (−6.1, 207.1)
Sensory organ diseases	7 (1, 13)	2	−5 (−11, 1)	−72.2 (−84.7, 50.5)
Musculoskeletal disorders	192 (154, 231)	188	−4 (−43, 34)	−2.3 (−18.5, 22.0)
Malignant neoplasms	6047 (5832, 6263)	6035	−12 (−228, 203)	−0.2 (−3.6, 3.5)
Nutritional deficiencies	338 (278, 398)	313	−25 (−85, 35)	−7.3 (−21.3, 12.6)
Dermatological diseases	377 (269, 485)	312	−65 (−173, 43)	−17.3 (−35.7, 16.0)
Mental and nervous system diseases	1337 (1242, 1431)	1256	−81 (−175, 14)	−6.0 (−12.2, 1.1)
Self-inflicted injuries (suicide)	711 (607, 815)	629	−82 (−186, 22)	−11.5 (−22.8, 3.6)
Digestive diseases	2659 (2543, 2775)	2562	−97 (−213, 19)	−3.6 (−7.7, 0.7)
Neonatal diseases	523 (437, 610)	443	−80 (−167, 6)	−15.3 (−27.4, 1.5)
Other neoplasm	632 (514, 750)	556	−76 (−194, 42)	−12.0 (−25.9, 8.2)
Infectious and parasitic diseases	1501 (1373, 1628)	1305	−196 * (−323, −68)	−13.0 * (−19.9, −4.9)
Non-infectious respiratory diseases	2285 (1982, 2588)	1793	−492 * (−795, −189)	−21.5 * (−30.7, −9.6)
Homicides	3864 (3121, 4607)	3424	−440 (−1183, 303)	−11.4 (−25.7, 9.7)
Events of undetermined intent	213 (71, 637)	430	217 (−207, 359)	101.8 (−32.5, 503.5)
Total	46,811 (45,592, 48,030)	61,293	14,482 (13,263, 15,701)	30.9 * (27.6, 34.4)

Source: Own elaboration based on data provided by the Epidemiological and Statistical Death Subsystem (SEED) of the State of Sonora for the period 2015–2022. † Deaths with a well-defined cause of death subgroup were counted, according to the Global Burden of Disease. * Statistically significant at the α = 0.05 level.

## Data Availability

The data used in this study are subject to institutional and regulatory restrictions and are not publicly available. However, they may be made available upon reasonable request and with appropriate authorization from the relevant health authorities. The data presented in this study are available on request from the corresponding author.

## Supplementary Materials

The following supporting information can be downloaded at: https://www.mdpi.com/article/10.3390/epidemiologia7040105/s1. Table S1: Information criteria comparing two model specifications for the secular trend; Figure S1: Time Series of model-based expected deaths and observed deaths by cause of death subgroup and ordered by total sum of deaths during the pre-pandemic period (ischemic heart disease, malignant neoplasms, diabetes and digestive diseases); Figure S2: Time Series of model-based expected deaths and observed deaths by cause of death subgroup and ordered by total sum of deaths during the pre-pandemic period (other cardiovascular disease, respiratory infections and tuberculosis, non-infectious respiratory diseases and cerebrovascular disease); Figure S3: Time Series of model-based expected deaths and observed deaths by cause of death subgroup and ordered by total sum of deaths during the pre-pandemic period (non-transport-related accidents, homicides, infectious and parasitic diseases and transport-related accidents); Figure S4: Time Series of model-based expected deaths and observed deaths by cause of death subgroup and ordered by total sum of deaths during the pre-pandemic period (genitourinary diseases, mental and nervous system diseases, neonatal diseases and hypertensive heart disease); Figure S5: Time Series of model-based expected deaths and observed deaths by cause of death subgroup and ordered by total sum of deaths during the pre-pandemic period (self-inflicted injuries [suicide], other neoplasm, other endocrine, metabolic, hematological and immunological diseases and nutritional deficiencies); Figure S6: Time Series of model-based expected deaths and observed deaths by cause of death subgroup and ordered by total sum of deaths during the pre-pandemic period (congenital defects, ill-defined causes, dermatological diseases and events of undetermined intent); Figure S7: Time Series of model-based expected deaths and observed deaths by cause of death subgroup and ordered by total sum of deaths during the pre-pandemic period (musculoskeletal disorders, maternal diseases and sensory organ diseases).

## Abbreviations

The following abbreviations are used in this manuscript:

EM	Excess mortality
MR	Mortality rate
SEED	Epidemiological and Statistical Death Subsystem
COVID-19	Coronavirus Disease of 2019
ICD-10	International Classification of Diseases, 10th. Revision
GBD	Global Burden of Disease
