# Supplementary material for: Excess Mortality During the COVID-19 Pandemic in Sonora, Mexico, 2015–2022: A Time-Series Analysis of Cause-Specific Mortality
Source: Epidemiologia (Basel). 2026 Jul 28;7(4):105. doi: 10.3390/epidemiologia7040105 (PMC13512150; doi:10.3390/epidemiologia7040105)
Supplement: Supplementary file 1 [file epidemiologia-07-00105-s001.zip › epidemiologia-4285079-supplementary.pdf]

Supplementary Material

Excess Mortality During the COVID-19 Pandemic in Sonora, Mexico, 2015–2022: A Time-Series Analysis of Cause-Specific Mortality

Diego I. Álvarez-López [1], Elizabeth Ferreira-Guerrero [2], Lina S. Palacio-Mejía [3], Amado D. Quezada-Sánchez [3], Jorge Laureano-Eugenio [4], Sergio Trujillo-López [5], Mariann Duarte-Badillo [5] and Gerardo Álvarez-Hernández [5,*]

[1] Department of Population and Public Health Sciences, Keck School of Medicine, University of Southern California, Los Angeles, CA 90032, USA; dialvare@usc.edu
[2] Centro de Investigación sobre Enfermedades Infecciosas, Instituto Nacional de Salud Pública, Cuernavaca 62100, Mexico; elizabeth.ferreira@insp.mx
[3] Centro de Investigación en Evaluación y Encuestas, Instituto Nacional de Salud Pública, Cuernavaca 62100, Mexico; lpalacio@insp.mx (L.S.P.-M.); amado.quezada@insp.mx (A.D.Q.-S.)
[4] Subsecretaría de Servicios de Salud Colectiva, Secretaría de Salud Pública del Estado de Sonora, Hermosillo 83000, Mexico; subserviciossaludcolectiva@gmail.com
[5] Departamento de Medicina y Ciencias de la Salud, Universidad de Sonora, Hermosillo 83000, Mexico; sergio.trujillo@unison.mx (S.T.-L.); a222214072@unison.mx (M.D.-B.)
* Correspondence: gerardo.alvarez@unison.mx; Tel.: +52-662-2592121

Table of Contents

Table S1. Information criteria comparing two model specifications for the secular trend 2

Figures S1-S7. Time Series of model-based expected deaths and observed deaths by cause of death subgroup and ordered by total sum of deaths during the pre-pandemic period............. 3

Table S1. Information criteria comparing two model specifications for the secular trend

	Akaike Information Criteria			Bayesian Information Criteria		
	Linear	Splines	Best fit	Linear	Splines	Best fit
Ischemic heart disease	840.6	844.6	Linear	868.2	876.5	Linear
Cerebrovascular disease	673.1	677.1	Linear	700.7	709.0	Linear
Hypertensive heart disease	529.5	533.5	Linear	557.2	565.4	Linear
Other cardiovascular diseases	691.7	695.6	Linear	719.4	727.5	Linear
Diabetes	769.0	773.0	Linear	796.6	804.9	Linear
Respiratory infections and tuberculosis	681.0	684.9	Linear	708.6	716.8	Linear
Non-transport-related accidents	658.7	662.7	Linear	686.3	694.6	Linear
Ill-defined causes	467.1	471.1	Linear	494.8	503.0	Linear
Other endocrine, metabolic, hematological, and immunological diseases	498.2	502.1	Linear	525.9	534.0	Linear
Transport-related accidents	600.4	604.3	Linear	628.1	636.2	Linear
Genitourinary diseases	598.4	602.3	Linear	626.0	634.2	Linear
Congenital defects	475.7	478.9	Linear	503.3	510.8	Linear
Maternal diseases	204.4	207.3	Linear	232.1	239.2	Linear
Sensory organ diseases	89.8	93.3	Linear	117.4	125.2	Linear
Musculoskeletal disorders	401.9	405.7	Linear	429.5	437.6	Linear
Malignant neoplasms	808.7	812.7	Linear	836.4	844.6	Linear
Nutritional deficiencies	485.4	489.2	Linear	513.0	521.1	Linear
Dermatological diseases	446.0	450.0	Linear	473.7	481.9	Linear
Mental and nervous system diseases	596.6	600.6	Linear	624.3	632.5	Linear
Self-inflicted injuries (suicide)	507.2	511.0	Linear	534.8	542.9	Linear
Digestive diseases	707.9	711.9	Linear	735.5	743.8	Linear
Neonatal diseases	530.2	534.0	Linear	557.8	566.0	Linear
Other neoplasm	503.7	507.4	Linear	531.3	539.3	Linear
Infectious and parasitic diseases	617.6	621.6	Linear	645.3	653.5	Linear
Non-infectious respiratory diseases	673.9	677.5	Linear	701.6	709.4	Linear
Homicides	650.3	653.6	Linear	678.0	685.5	Linear
Events of undetermined intent	406.8	400.2	Splines	434.5	432.1	Splines

Figures S1. Time Series of model-based expected deaths and observed deaths by cause of death subgroup and ordered by total sum of deaths during the pre-pandemic period (ischemic heart disease, malignant neoplasms, diabetes and digestive diseases)

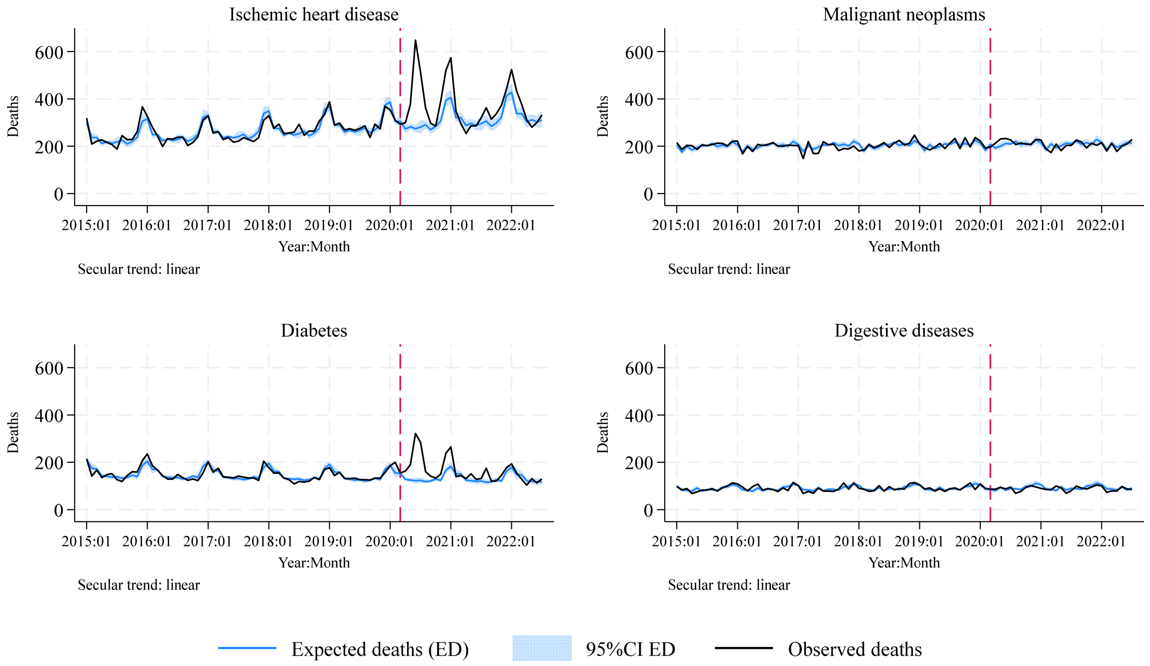

Figure S2: Time Series of model-based expected deaths and observed deaths by cause of death sub-group and ordered by total sum of deaths during the pre-pandemic period (other cardiovascular disease, respiratory infections and tuberculosis, non-infectious respiratory diseases and cerebrovascular disease)

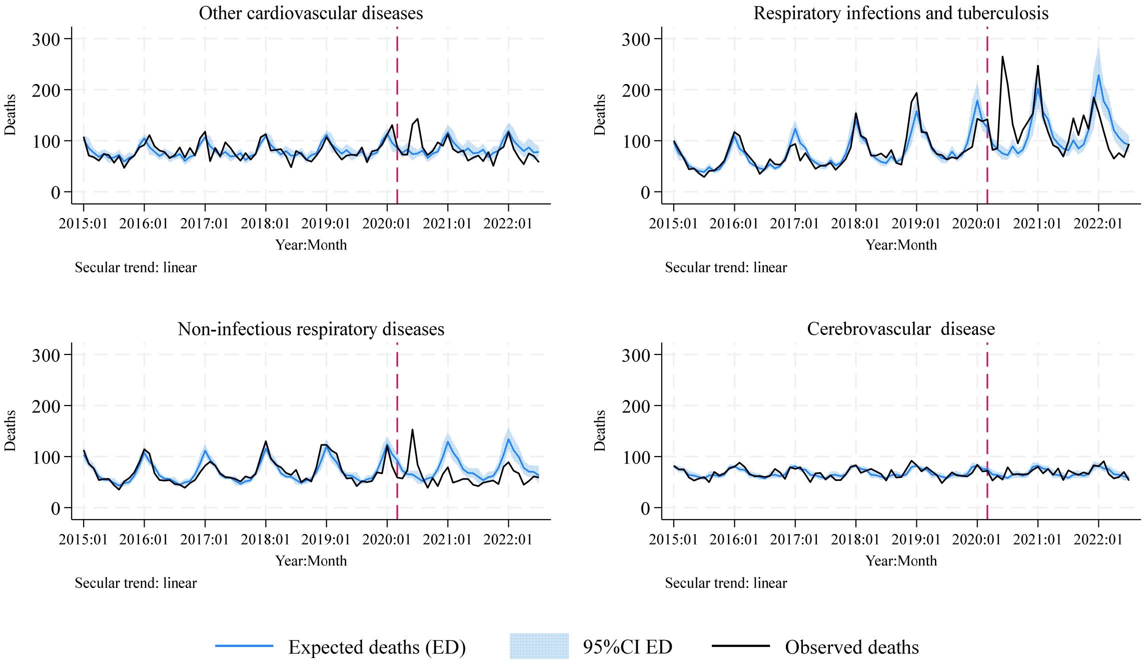

Figure S3: Time Series of model-based expected deaths and observed deaths by cause of death sub-group and ordered by total sum of deaths during the pre-pandemic period (non-transport-related accidents, homicides, infectious and parasitic diseases and transport-related accidents)

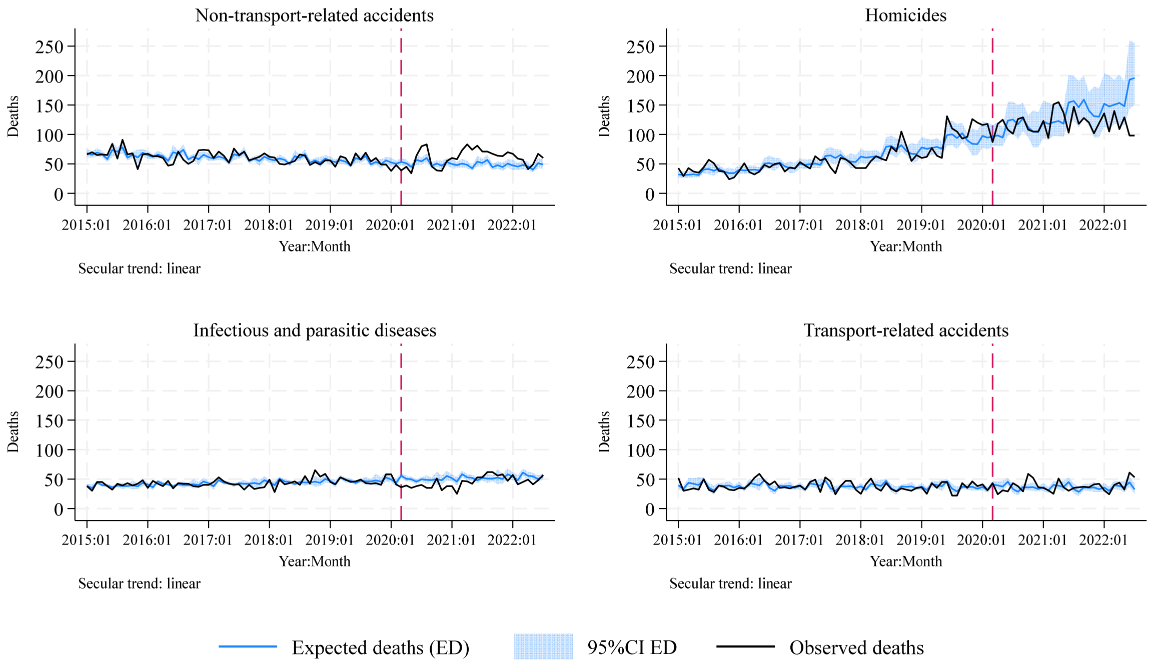

Figure S4: Time Series of model-based expected deaths and observed deaths by cause of death sub-group and ordered by total sum of deaths during the pre-pandemic period (genitourinary diseases, mental and nervous system diseases, neonatal diseases and hypertensive heart disease)

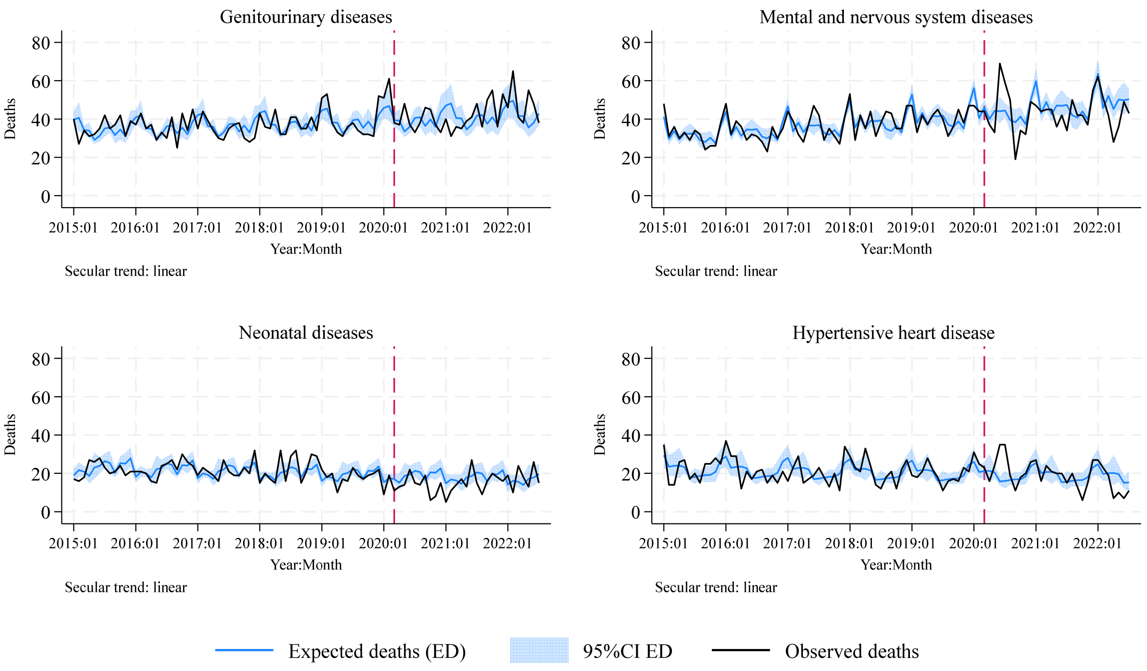

Figure S5: Time Series of model-based expected deaths and observed deaths by cause of death sub-group and ordered by total sum of deaths during the pre-pandemic period (self-inflicted injuries [suicide], other neoplasm, other endocrine, metabolic, hematological and immunological diseases and nutritional deficiencies)

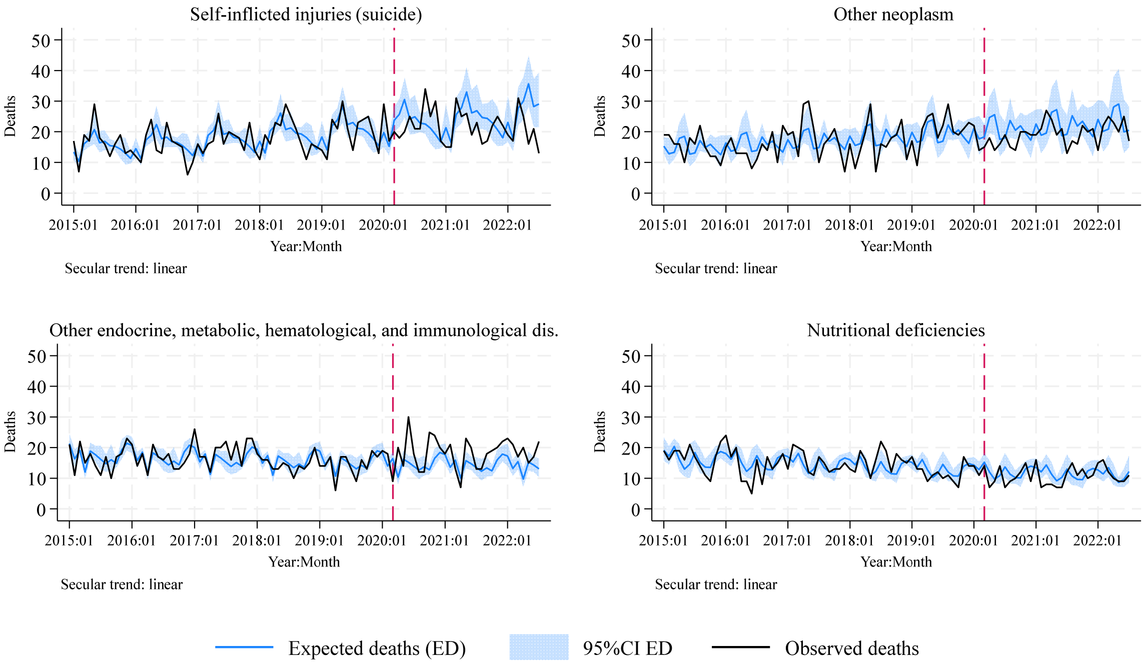

Figure S6: Time Series of model-based expected deaths and observed deaths by cause of death sub-group and ordered by total sum of deaths during the pre-pandemic period (congenital defects, ill-defined causes, dermatological diseases and events of undetermined intent)

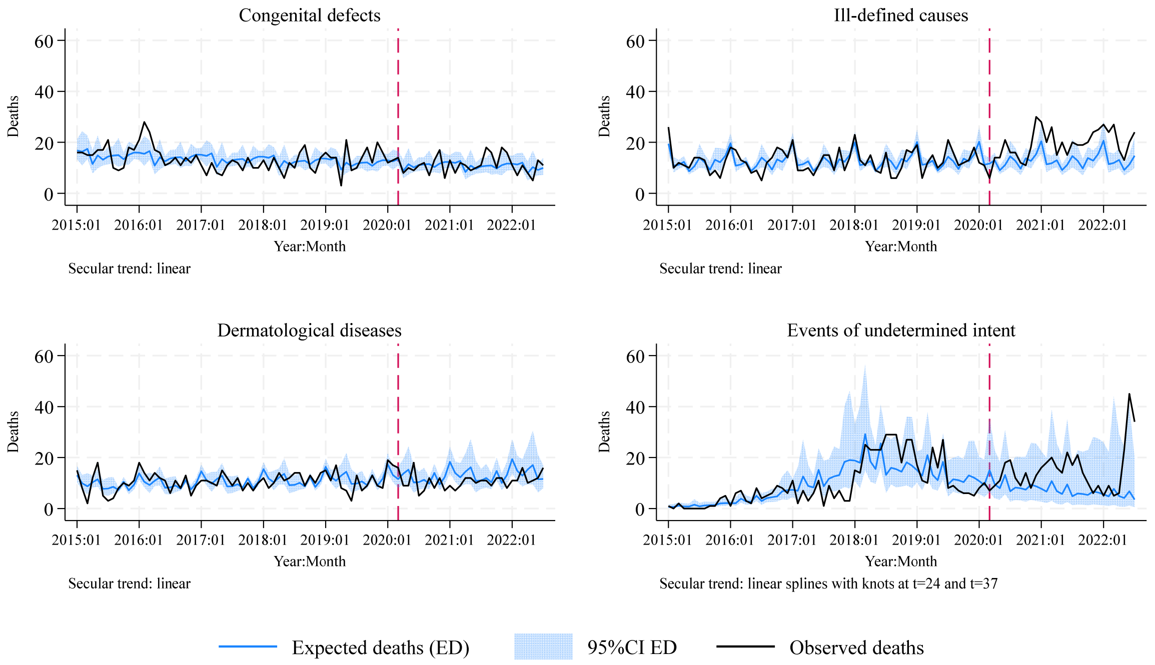

Figure S7: Time Series of model-based expected deaths and observed deaths by cause of death sub-group and ordered by total sum of deaths during the pre-pandemic period (musculoskeletal disorders, maternal diseases and sensory organ diseases).

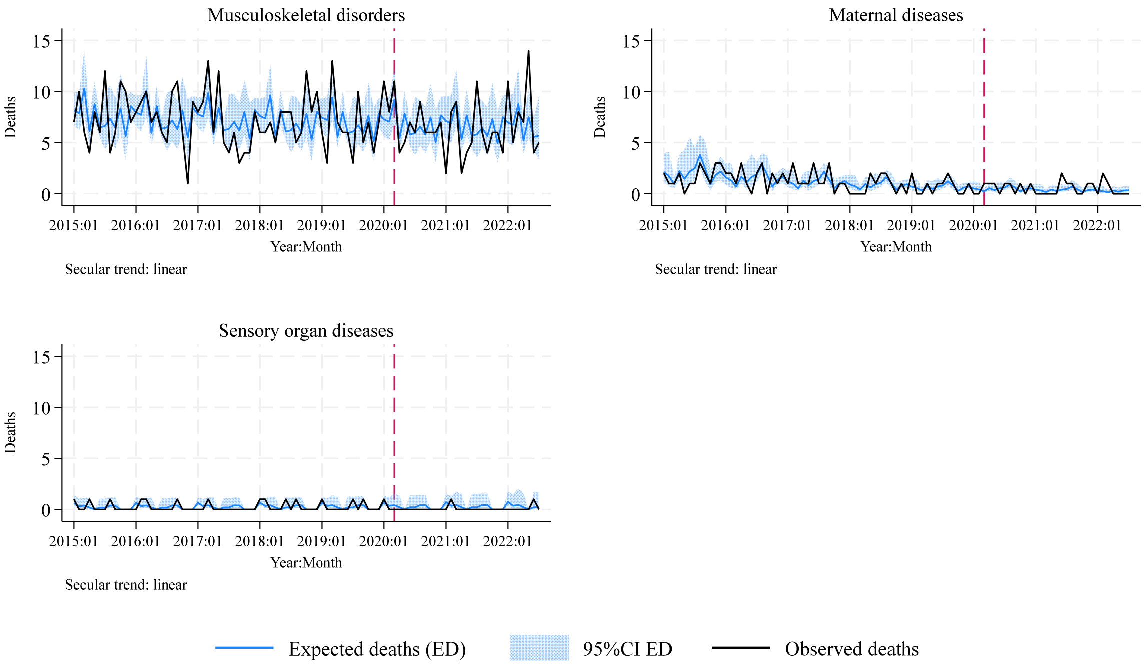

The red dashed line marks the starting month of the COVID-19 pandemic in Mexico (March 2020).
